# Early Machine Learning-Based Identification of Hospitalized Patients at Low Risk of Respiratory Deterioration or Mortality in Community-Acquired Pneumonia: External Validation of a Multivariable Model

**DOI:** 10.3390/idr18040077

**Published:** 2026-07-25

**Authors:** Claudia Gyimah, Prasamsa Pudasaini, Allison LeMahieu, Phillip Schulte, Yewande E. Odeyemi

**Affiliations:** 1 Division of Pulmonary and Critical Care Medicine, Mayo Clinic, 200 First Street SW, Rochester, MN 55905, USA; gyimah.claudia@mayo.edu (C.G.);; 2 Division of Clinical Trials and Biostatistics, Mayo Clinic, Rochester, MN 55905, USA

**Keywords:** community-acquired pneumonia, machine learning models, hospitalized patients, respiratory deterioration, and mortality

## Abstract

Background/Objective: To externally validate our previously published machine learning model for identifying hospitalized patients with community-acquired pneumonia (CAP) at low risk of respiratory deterioration or death using the Medical Information Mart for Intensive Care IV (MIMIC-IV) database. Methods: This is a retrospective cohort study of adult patients (≥18 years) who were admitted with CAP and acute hypoxemic respiratory failure using the publicly available MIMIC-IV critical care dataset (Beth Israel Deaconess Medical Center, Boston, MA). We conducted an external validation study of a previously developed gradient boosting machine (GBM) model without recalibration using data available within the first 6 h of hospital admission. Results: For the primary composite outcome (need for advanced respiratory support [high flow nasal cannula (HFNC), non-invasive mechanical ventilation (NIMV), invasive mechanical ventilation (IMV)] or in-hospital death), the gradient boosting model demonstrated comparable performance in the derivation and external validation cohorts. The area under the receiver operating characteristic curve (AUC) was 0.713 in the Mayo cohort (n = 4379) and 0.689 in the MIMIC-IV cohort. Accuracy was 0.612 (95% confidence interval [CI], 0.595–0.628) versus 0.606 (95% CI 0.600–0.611), specificity 0.574 versus 0.523, sensitivity 0.723 versus 0.754, NPV 0.860 versus 0.842, and PPV 0.364 versus 0.401, respectively. For secondary outcomes, model discrimination was comparable between cohorts. The AUC for in-hospital mortality was 0.727 in the Mayo Cohort versus 0.733 in MIMIC-IV; for IMV, 0.736 versus 0.724; and for NIMV, 0.732 versus 0.708. Conclusions: Our machine learning algorithm demonstrated good discrimination and a high negative predictive value for identifying low-risk hospitalized CAP patients for respiratory deterioration or death in the external MIMIC-IV dataset, supporting its potential utility for prognostic enrichment in pneumonia clinical trials.

## 1. Introduction

Community-acquired pneumonia (CAP) remains a major cause of hospitalizations among adults and is associated with substantial morbidity, mortality, and healthcare costs worldwide [1,2]. Despite advancements in antimicrobial therapy, vaccination, and supportive care, CAP remains a leading cause of sepsis, acute respiratory failure, and intensive care unit (ICU) admissions [3,4]. The burden is particularly pronounced among individuals with advanced age, chronic lung disease, chronic heart disease, cardiovascular disease, diabetes mellitus, malnutrition, immunocompromising conditions, and those with lifestyle factors such as smoking and excessive alcohol consumption [5,6]. A 2024 review noted that in the United States alone, CAP accounted for approximately 1.4 million emergency department visits, 740,000 hospitalizations, 41,000 deaths, and $7.7 billion in inpatient costs annually [7].

Against this backdrop of high and variable morbidity, the clinical trajectories of hospitalized CAP patients are notably heterogeneous. While many hospitalized patients recover with standard oxygen therapy and antimicrobial treatment, a clinically important subset experiences rapid deterioration requiring escalation to advanced respiratory support, including high-flow nasal cannula (HFNC), non-invasive mechanical ventilation (NIMV), invasive mechanical ventilation (IMV), vasopressor support, or ICU transfer [8,9,10,11]. Early recognition of patients at risk of clinical deterioration remains challenging because physiological instability may emerge after initial presentation despite seemingly reassuring admission findings [10]. Patients may deteriorate rapidly, sometimes within the first hours of hospitalization, making early and reliable identification of high-risk individuals paramount for guiding clinical decisions and allocating resources appropriately [10,12].

Several conventional severity tools are widely used in CAP, most notably the Pneumonia Severity Index (PSI) and confusion, uremia, respiratory rate, blood pressure, and age ≥ 65 years (CURB-65). These instruments were designed to predict mortality risk and inform disposition decisions such as outpatient versus inpatient management rather than the need for advanced oxygen strategies, ICU admission, or organ support, making them suboptimal for identifying patients at risk of deterioration [6,10]. Although both scores have demonstrated reasonable discriminatory performance for short-term mortality, they share fundamental limitations in predicting the dynamic clinical trajectory of hospitalized patients. Specifically, neither tool captures the risk of acute respiratory deterioration during hospitalization—a clinically critical endpoint that is distinct from mortality and often precedes it [13]. Furthermore, these tools were derived predominantly from pre-ICU era cohorts and may not reflect contemporary patterns of care, including the widespread adoption of high-flow nasal cannula and non-invasive ventilation [8,14].

In comparative studies, the Sequential Organ Failure Assessment (SOFA) score has been found most effective at predicting the need for ICU admission, though it often identifies organ failure already existing at the time of evaluation [10]. The American thoracic society/Infectious disease society of America (ATS/IDSA) recommends using the IDSA/ATS 2007 minor severity criteria together with clinical judgment to guide the need for ICU-level care in patients not immediately requiring vasopressors or mechanical ventilation [15]. The 2026 Surviving Sepsis Campaign guidelines recommend the use of screening tools such as the National Early Warning Score (NEWS, NEWS2), Modified Early Warning Score (MEWS), or Systemic Inflammatory Response Syndrome (SIRS) for sepsis identification in hospitalized patients, based on consistent evidence that early warning scores demonstrate higher sensitivity for detecting clinical deterioration [16]. Although these tools are useful for identifying patients at low risk of mortality, they were not developed to predict respiratory deterioration and do not reliably identify patients who will subsequently require advanced respiratory support. Delayed recognition of patients who ultimately require escalation of respiratory support has been associated with worse outcomes, including increased mortality and prolonged hospitalization [17]. These limitations highlight a critical gap in current risk stratification approaches.

Improved prognostic tools have the potential to enhance both clinical care and clinical research. In routine practice, accurate early risk stratification may facilitate timely monitoring, escalation of care, and resource allocation. In addition, reliable identification of patients at high risk of respiratory deterioration can support prognostic enrichment in clinical trials by selecting patients most likely to experience clinically relevant endpoints, thereby improving trial efficiency and reducing sample size requirements [18]. These applications are complementary but serve distinct clinical and research objectives. Machine learning approaches may offer advantages over traditional point-based clinical scores [19]. Unlike linear additive systems, machine learning algorithms can capture nonlinear relationships, interactions among variables, and complex patterns within routinely collected electronic health record data [20]. Over the past decade, multiple machine learning models have been proposed for sepsis, deterioration detection, ICU triage, and mortality prediction [21,22]. While these tools show significant potential, most require further external validation before widespread clinical adoption.

We previously developed a gradient boosting machine model using variables available within the first six hours of hospital presentation to identify patients with CAP at low risk for respiratory deterioration or death [23]. In the derivation cohort, the model demonstrated moderate discrimination with strong negative predictive value, supporting potential use as a rule-out tool (area under the receiver operating characteristic curve [AUC] 0.713; sensitivity 0.723; negative predictive value [NPV] 0.860) [23]. Such a tool could have direct bedside utility by identifying patients unlikely to require escalation of care and informing early clinical decision-making. Beyond its potential clinical application, accurate risk prediction may also facilitate prognostic enrichment in clinical trials by identifying patients at greater risk for clinically meaningful outcomes. It may also offer research value by enabling prognostic enrichment in clinical trials through the exclusion of individuals unlikely to reach measurable endpoints. The primary objective of the present study was to externally validate the performance of this model in an independent cohort using the Medical Information Mart for Intensive Care IV (MIMIC-IV) database. As part of this validation, we assessed the model’s transportability (generalizability) and its ability to accurately identify patients at low risk for respiratory deterioration or death. We hypothesized that model performance would remain clinically meaningful in an independent cohort despite expected attenuation in discrimination.

## 2. Materials and Methods

### 2.1. Study Design and Data Source

We conducted an observational retrospective cohort study to externally validate a previously developed gradient boosting machine (GBM) model using the MIMIC-IV database. MIMIC-IV is a large, publicly available, de-identified database containing detailed electronic health record data from patients admitted to Beth Israel Deaconess Medical Center, including demographics, diagnoses, laboratory measurements, and clinical interventions [24,25]. Use of the database was approved under the relevant data use agreements, and the study was exempt from institutional review board oversight due to the use of de-identified data.

### 2.2. Study Population

Adult patients (≥18 years) hospitalized with CAP and acute hypoxemic respiratory failure were identified using methods aligned with the derivation cohort. Inclusion criteria for CAP were ICD diagnosis of pneumonia [International Classification of Diseases (ICD) 9 codes 481–486 and ICD 10 codes J13, J15, J18] and chest radiology report indicating infiltrate. Exclusion criteria were aspiration pneumonia, hospital-acquired pneumonia, ventilator-acquired pneumonia, interstitial lung disease, leukopenia, neutropenia, acquired immunodeficiency syndrome (AIDS), and human immunodeficiency virus (HIV). To preserve the intended prediction window and ensure comparability with the original model, we excluded patients who required advanced respiratory support—defined as high-flow nasal cannula (HFNC), non-invasive mechanical ventilation (NIMV), or invasive mechanical ventilation (IMV)—or who died within the first 6 h of presentation [23].

### 2.3. Predictor Variables

Predictor variables were selected a priori based on the original model and included data available within the first 6 h of hospital admission [23]. These variables comprised:

Demographics: age, sex, and race (categorized as White vs. other/unknown).

Comorbidities: congestive heart failure, chronic obstructive pulmonary disease, asthma, chronic liver disease, chronic kidney disease, and active or prior neoplasia.

Physiologic and laboratory data: vital signs and laboratory values, including white blood cell count with differential, serum bicarbonate, sodium, and blood urea nitrogen.

When multiple values were available within the 6 h window, the earliest recorded value was used to reflect real-time clinical decision-making.

### 2.4. Outcomes

The primary outcome was a composite of need for advanced respiratory support (HFNC, NIMV, or IMV) or in-hospital mortality.

Secondary outcomes comprised the individual components to assess model performance across each clinically important endpoint independently and included:

- In-hospital mortality
- Requirement for invasive mechanical ventilation (IMV)
- Requirement for non-invasive mechanical ventilation (NIMV)

### 2.5. Model Application

The previously developed GBM model was applied to the external cohort without retraining to assess generalizability. Predicted probabilities were generated for each patient, and a predefined probability threshold of >0.30 was used to classify patients as high risk. This threshold was selected in the derivation study to maximize sensitivity and achieve a NPV of at least 0.85, consistent with the model’s intended use as a screening tool to identify patients at low risk of clinical deterioration for potential prognostic enrichment in clinical trials.

### 2.6. Statistical Analysis

**Figure 1:**
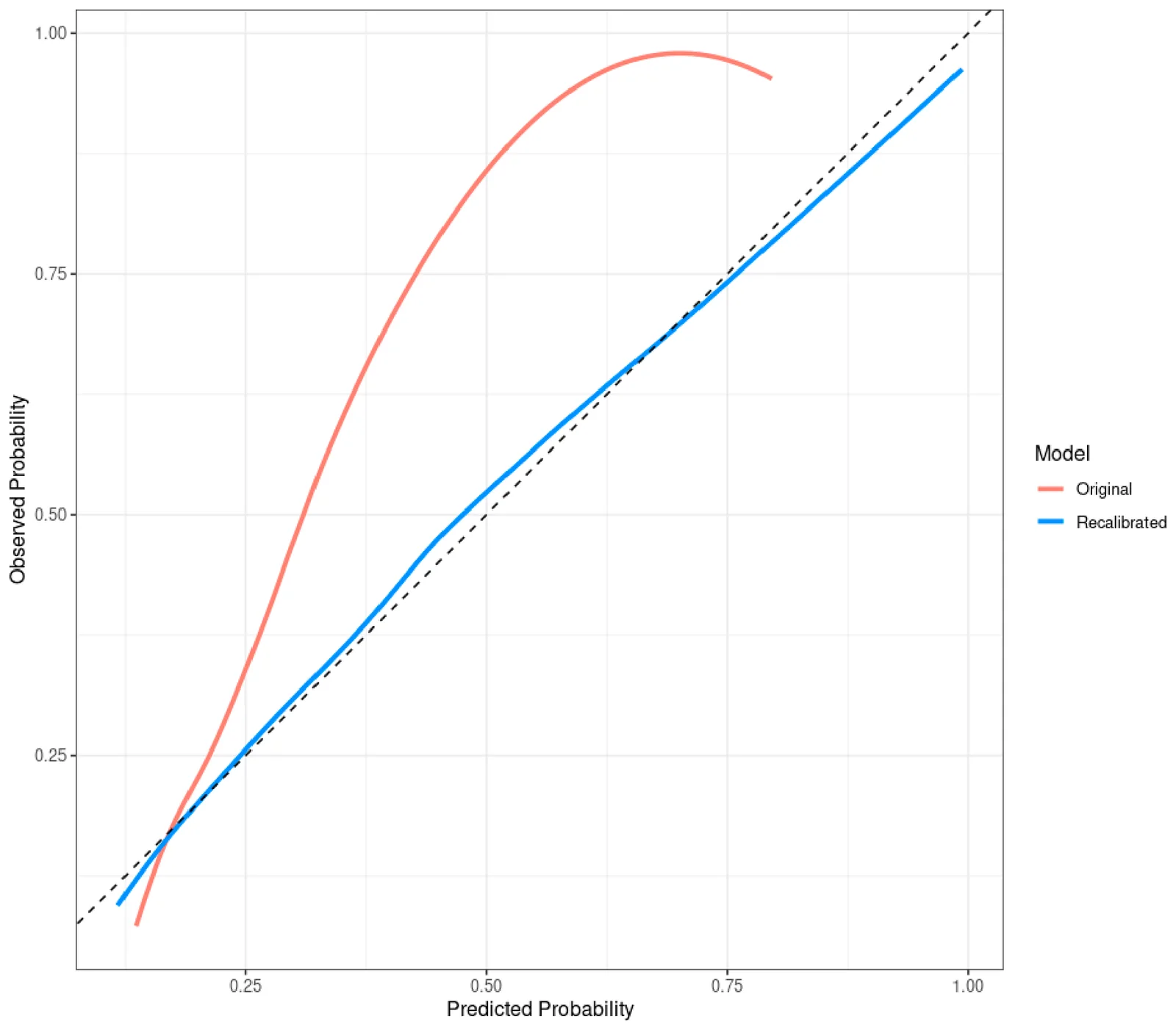
Calibration plot.

Patient characteristics were summarized using the median [interquartile range (IQR)] for continuous variables and the number (percentage) for categorical variables. These characteristics were summarized for the external validation cohort and the derivation cohort. Model performance was evaluated using standard measures of discrimination and classification. Discrimination was assessed using the AUC. Classification performance metrics included accuracy, sensitivity, specificity, NPV, and positive predictive value (PPV). Further, 95% binomial confidence intervals were calculated for all classification performance metrics. Calibration was assessed by comparing predicted and observed event rates across risk strata; see Figure 1. We observed poor calibration in our model when applied to these external validation data, with the model over-estimating risk at the low end and over-estimating risk among high-risk individuals. We performed a logistic recalibration, which showed good performance [26]. Additionally, calibration was assessed by subgroups of interest (age < 65 vs. age ≥ 65, congestive heart failure [CHF] yes/no, and chronic obstructive pulmonary disease [COPD] yes/no). A decision curve analysis was conducted to evaluate the prediction model. Any missing predictors were treated as a distinct and possibly informative segment of the data, reflecting actual practices where such omissions are common. This allows the resulting GBM prediction model to handle missing (unmeasured) inputs and still produce the predicted probability of an event. There were no missing data for the primary and secondary outcomes or for the following predictors: age, gender, or comorbidities. Data management and analysis were conducted using R version 4.1.2 (RStudio Team 2021, Boston, MA, USA). We used the R packages “gbm” and “caret” for model training.

## 3. Results

In the MIMIC-IV cohort (n = 5438), a total of 4622 patients (85.0%) were free of the primary outcome within the first 6 h of presentation and were therefore included in the validation analysis. Among these patients, 1459 (31.6%) subsequently experienced the primary composite outcome of requiring advanced respiratory support or in-hospital death. This event rate was higher than that observed in the derivation (Mayo) cohort, where 890 of 3528 patients (25.2%) met the primary outcome [23], suggesting a population with greater baseline severity or differing practice patterns in the external dataset (Table 1).

At the prespecified probability threshold of ≥0.30 [23], the GBM model demonstrated modest discrimination for the primary outcome, with comparable performance across cohorts (AUC 0.689 in MIMIC vs. 0.713 in Mayo). In the MIMIC cohort, sensitivity was 0.754 (vs. 0.723 in the Mayo cohort), indicating that the model identified approximately three-quarters of patients who went on to experience the outcome, while specificity was 0.523 (vs. 0.574 in the Mayo cohort), reflecting moderate ability to correctly identify those who remained event-free. The NPV remained high at 0.842 (vs. 0.860 in the Mayo cohort), consistent with the model’s intended role as a screening tool to identify low-risk patients. The PPV was 0.401 (vs. 0.364 in the Mayo cohort), and overall accuracy was 0.606 (95% confidence interval [CI], 0.600–0.611), similar to that observed in the Mayo cohort (accuracy 0.612 [95% CI, 0.595–0.628]). The calibration plot (Figure 1) indicates we are over-predicting the risk among low-risk patients and under-predicting risk for high-risk patients. For example, we predict 40% risk when the actual risk for that type of patient is closer to 70%. However, after recalibration, the plot indicates a similar prediction of risk among all risk levels. Subgroup calibration plots by age group, CHF history, and COPD history indicated similar calibration by group, indicating these key factors were not influential on calibration (Supplementary Figures S1–S3). Also, see the figure for variable importance in the external cohort (Supplementary Figure S4).

For the secondary outcome of in-hospital mortality, which occurred in 5.1% of patients in the MIMIC cohort compared with 3.5% in the Mayo cohort [22], model discrimination was similar between datasets (AUC 0.733 vs. 0.727). At the predefined threshold, specificity was very high in both cohorts (0.996 in MIMIC vs. 0.995 in Mayo), indicating that the model rarely classified survivors as high risk for death. However, sensitivity was low (0.074 vs. 0.077), reflecting limited ability to identify patients who ultimately died. Despite this, the NPV remained high (0.970 vs. 0.967), and overall accuracy was 0.971 (95% CI, 0.968–0.974) in MIMIC compared with 0.963 (95% CI, 0.957–0.968) in the Mayo Cohort.

For the outcome of need for invasive mechanical ventilation (IMV), the model demonstrated moderate discrimination (AUC 0.724 in MIMIC versus 0.736 in Mayo). Sensitivity was relatively low (0.347 vs. 0.321), indicating that a substantial proportion of patients requiring IMV were not identified at the chosen threshold. However, the NPV remained high (0.911 vs. 0.924), suggesting that patients classified as low risk were unlikely to require invasive ventilation. For the need for non-invasive mechanical ventilation (NIMV), discrimination was similarly moderate (AUC 0.708 in MIMIC vs. 0.732 in Mayo), with sensitivity of 0.636 vs. 0.619, and NPV of 0.880 vs. 0.892.

Overall, model performance in the external validation cohort was consistent with that observed in the derivation cohort, with stable discrimination and preservation of high negative predictive value across outcomes, supporting its potential utility as a rule-out tool for identifying patients at low risk of early respiratory deterioration.

Complete performance metrics are provided in Table 2.

**Figure 2:**
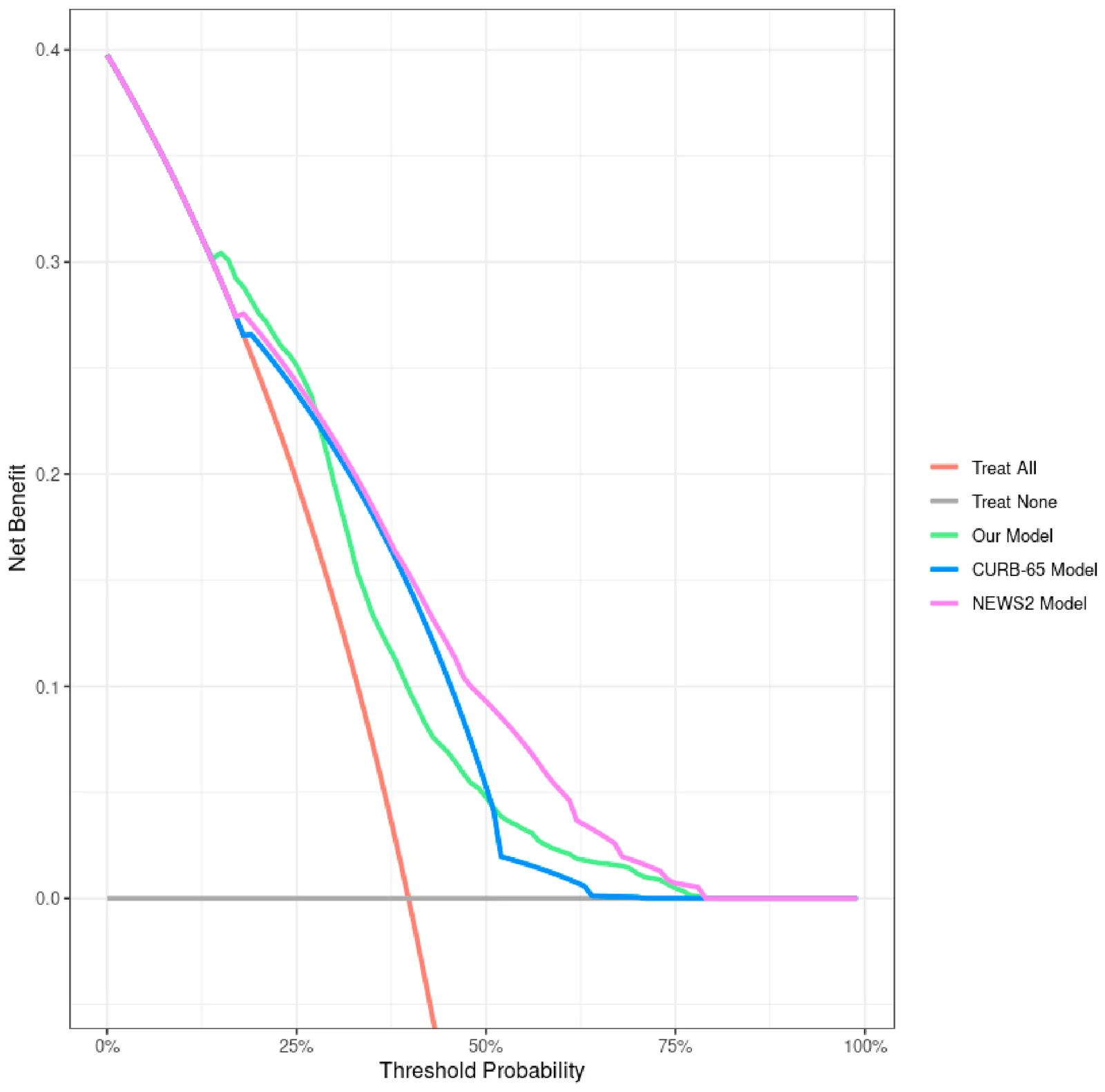
Decision curve analysis.

Decision curve analysis (Figure 2) suggested a range of threshold probabilities for which our model could yield higher net benefit than default treat-none or treat-all strategies. Compared to CURB-65 and NEWS2, some threshold probabilities (<median in our data) are consistent with higher net benefit for our model, whereas higher threshold probabilities align with higher net benefit for comparator models.

## 4. Discussion

In this external verification with the MIMIC-IV database, our GBM prognostic model demonstrated preserved and clinically meaningful performance as a rule-out tool for early identification of hospitalized patients with CAP who are at low risk of respiratory deterioration or death using data available within the first six hours of admission. Although the AUC for the primary outcome declined modestly from 0.713 to 0.689, this degree of performance attenuation is expected in external validation settings and is consistent with prior studies evaluating the transportability of clinical prediction models [26]. External validation often exposes optimism in derivation-phase estimates, as models are inherently calibrated to the source population; variations in demographics, comorbidity burden, documentation practices, oxygen delivery pathways, ICU admission thresholds, and respiratory support preferences can all affect transportability [27,28]. The MIMIC-IV cohort represents a clinically distinct environment compared with the original health system, making preserved performance particularly encouraging. Importantly, sensitivity increased from 0.723 to 0.754, aligning with the model’s intended design to minimize false negatives, which is critical for safely identifying low-risk patients. In the context of a rule-out tool, high sensitivity ensures that patients at genuine risk of deterioration are not incorrectly classified as low risk and potentially undertreated. The observed reduction in specificity (0.574 to 0.523) likely reflects systematic differences in disease severity and care practices between the derivation and validation cohorts; MIMIC-IV is an ICU-enriched dataset from a large academic tertiary center, and patients in this cohort may have had more severe presentations at baseline. Despite this, the NPV remained high at 0.842, reinforcing the model’s utility as a rule-out tool for clinical and research purposes.

Performance across secondary endpoints further reinforces the model’s robustness and cross-population stability. Discrimination for in-hospital mortality improved slightly in the MIMIC cohort (AUC 0.727 to 0.733), while predictive performance for invasive mechanical ventilation (AUC 0.736 to 0.724) and noninvasive ventilation (AUC 0.732 to 0.708) remained stable across sites. These findings suggest that the model captures clinically relevant signals of disease severity early in the course of illness and maintains consistent performance across a spectrum of respiratory support outcomes. The high NPV for in-hospital mortality (0.970 in MIMIC) is particularly noteworthy, as it suggests that patients classified as low-risk by the model have a very high probability of surviving to hospital discharge without requiring intubation or dying. These performance characteristics are well aligned with a prognostic enrichment strategy, wherein patients categorized as low risk are excluded to increase event rates and improve the trial’s ability to detect treatment effects. Notably, the ability to predict respiratory deterioration using only early admission data is particularly valuable in CAP, where clinical trajectories can evolve rapidly, and early risk stratification remains challenging. Because the model relies solely on routinely collected clinical variables available early after admission, it has the potential to be integrated into existing clinical workflows. However, prospective implementation studies are needed to determine its impact on clinical decision-making and patient outcomes. Moreover, the consistent discriminatory performance across multiple secondary outcomes indicates that the model captures true physiologic risk signals rather than overtraining to a single outcome definition.

A key strength of this study is the strict application of the original model without recalibration or retraining, providing a rigorous assessment of model transportability. This approach represents a higher standard of external validation and avoids optimistic bias associated with model updating [27,28]. By applying the GBM model in its original form to an entirely independent cohort with different patient demographics, EHR infrastructure, and institutional care pathways, we provide strong evidence of its generalizability. In addition, the use of a large, publicly available ICU dataset enhances reproducibility and allows other investigators to independently verify and extend our results. The model’s reliance on early admission variables that are routinely available in most inpatient settings- including vital signs, basic laboratory results, comorbidity data- further supports its feasibility for real-world implementation without the need for custom data integration infrastructure. supports the feasibility of future implementation and prospective evaluation without requiring specialized biomarkers or custom data integration infrastructure.

These results have important clinical and research implications. From a clinical perspective, the model has potential utility as a rule-out tool to identify hospitalized patients with CAP who are unlikely to experience respiratory deterioration or death. If confirmed in prospective implementation studies, this information could complement clinical judgment by informing monitoring intensity, triage decisions, and resource allocation. Importantly, the model is intended to supplement rather than replace existing clinical assessment and should not be interpreted as a general severity score. From a research standpoint, the model may serve as a prognostic enrichment tool by identifying patients unlikely to experience key endpoints such as respiratory failure or death, thereby increasing event rates and improving trial efficiency [17,29]. This application is particularly relevant for CAP trials evaluating immunomodulatory therapies or respiratory support strategies, where heterogeneity in disease severity may dilute treatment effects [29,30]. Accordingly, incorporating a validated machine learning–based enrichment tool into CAP trial design may help accelerate the development and regulatory assessment of new therapies for this high-burden disease.

Despite these strengths, several limitations warrant consideration. As with all observational datasets, differences in data capture, coding practices, and treatment patterns across institutions may influence model performance and introduce unmeasured confounding. We acknowledge that race was dichotomized as White versus other/unknown in the original model because of the available data and sample size during model development. This simplification does not capture the heterogeneity of racial and ethnic groups and may limit assessment of model performance across underrepresented populations. Future work should evaluate model performance across more granular racial and ethnic categories and assess algorithmic fairness using diverse external cohorts. We also acknowledge that MIMIC-IV represents patients from a single tertiary academic medical center. Although MIMIC-IV provides an independent external validation cohort, validation in additional health systems and community hospitals with differing patient populations, practice patterns, and resource availability will be important to further establish the model’s transportability and generalizability. In addition, the model was validated primarily in an ICU population, which may limit generalizability to general medical ward settings where a substantial proportion of hospitalized CAP patients are managed. Finally, although the model uses variables available within the first six hours of admission, the clinical impact of integrating the model into real-time workflows has not been prospectively evaluated. Accordingly, prospective implementation studies are needed before routine clinical use can be recommended.

Altogether, these limitations define a clear agenda for future research. Prospective, multicenter studies are needed to determine whether use of the model improves clinical decision-making, resource utilization, or patient outcomes. These studies should also evaluate calibration across diverse healthcare settings and patient populations before consideration of routine clinical adoption. Also, prospective studies evaluating model performance in non-ICU ward settings are particularly important, as most hospitalized CAP patients are initially managed outside the ICU. Such studies will also be critical to establishing the role of this model in enabling prognostic enrichment in pneumonia-specific clinical trials by identifying and excluding low-risk patients unlikely to benefit from intervention.

## 5. Conclusions

We externally validated the GBM model for early risk stratification of hospitalized CAP patients using the MIMIC-IV critical care cohort. The model maintained discriminatory performance in an independent population, supporting its generalizability and utility as an early rule-out tool for patients at low risk for respiratory deterioration and/or death. These findings strengthen the evidence for its use as a prognostic enrichment tool for pneumonia clinical trials and warrant prospective multicenter evaluation in real-world clinical and trial settings.

## Figures and Tables

**Table 1:** Patient characteristics per cohort.

Characteristic	MIMIC-IV Cohort (n = 5438)	Mayo Cohort (n = 4379)
Sex, n (%)		
Female	2667 (49)	2015 (46)
Male	2771 (51)	2364 (54)
Age (years), median (IQR)	71.9 (61.0, 82.4)	73.6 (61.2, 83.3)
Race, n (%)		
American Indian/Alaskan Native	38 (1)	19 (0)
Asian	256 (5)	51 (1)
Black or African American	712 (13)	67 (2)
Other	446 (8)	85 (2)
Unknown	54 (1)	28 (1)
White	3932 (72)	4129 (94)
Comorbidities, n (%)		
Asthma	935 (17)	664 (15)
Congestive heart failure	1327 (24)	1334 (30)
First physical examination, median (IQR)		
Altered mental status, n (%)	696 (13)	534 (12)
Weight (kg)	77.6 (63.7, 94.8)	78.7 (64.7, 95.6)
Diastolic BP (mmHg)	66.0 (57.0, 80.0)	69.0 (59.0, 81.0)
Systolic BP (mmHg)	125.0 (112.0, 140.0)	127.0 (112.0, 144.0)
Pulse (bpm)	92.0 (78.0, 106.0)	91.0 (78.0, 105.0)
Respiratory rate (rpm)	20.0 (18.0, 23.5)	21.0 (18.0, 25.0)
Temperature (°C)	36.3 (36.0, 37.1)	36.8 (36.6, 37.2)
Lab and radiologic findings, median (IQR)		
Blood urea nitrogen (mg/dL)	22.0 (15.0, 30.0)	20.0 (14.0, 30.0)
Glucose(mg/dL)	133.0 (110.0, 164.0)	129.0 (108.0, 165.0)
Hematocrit (%)	36.3 (30.9, 40.8)	37.5 (33.3, 41.2)
Sodium (mmol/L)	134.0 (132.0, 141.0)	137.0 (134.0, 140.0)
WBC (×10^9^/L)	11.9 (8.9, 16.0)	11.8 (8.6, 15.8)
Neutrophils (×10^9^/L)	9.2 (6.5, 13.1)	9.5 (6.6, 13.2)
Eosinophils (×10^9^/L)	0.1 (0, 0.2)	0.1 (0, 0.2)
Lymphocytes (×10^9^/L)	1.0 (0.7, 1.5)	1.0 (0.6, 1.5)
Neutrophil/Lymphocyte ratio	9.9 (5.5, 16.7)	9.7 (5.4, 17.0)
Bicarbonate (mmol/L)	25.0 (23.0, 27.0)	25.0 (23.0, 28.0)

**Table 2:** GBM Model Performance in Mayo and MIMIC-IV Cohorts.

Outcome	Cohort	AUC (95% CI)	Accuracy (95% CI)	Spec. (95% CI)	Sens. (95% CI)	NPV (95% CI)	PPV (95% CI)
Advanced resp. support or mortality	Mayo ^1^	0.713 (0.693–0.732)	0.612 (0.595–0.628)	0.574 (0.555–0.593)	0.723 (0.690–0.750)	0.860 (0.842–0.875)	0.364 (0.341–0.386)
	MIMIC	0.689 (0.676–0.700)	0.606 (0.600–0.611)	0.523 (0.511–0.535)	0.754 (0.742–0.767)	0.842 (0.830–0.852)	0.401 (0.387, 0.414)
	MIMIC-CURB-65	0.696 (0.681–0.711	0.655 (0.641–0.669)	0.538 (0.519–0.557)	0.833 (0.815–0.850)	0.830 (0.811–0.847)	0.543 (0.524–0.562)
	MIMIC-NEWS2	0.746 (0.731–0.760)	0.656 (0.641–0.670)	0.527 (0.508–0.546)	0.851 (0.818–0.853)	0.842 (0.824–0.860)	0.543 (0.524–0.562)
Mortality alone	Mayo ^1^	0.727 (0.688–0.778)	0.963 (0.957–0.968)	0.995 (0.992–0.997)	0.077 (0.045–0.014)	0.967 (0.961–0.972)	0.364 (0.352–0.378)
	MIMIC	0.733 (0.720–0.749)	0.971 (0.968–0.974)	0.996 (0.994–0.998)	0.074 (0.069–0.080)	0.970 (0.965–0.973)	0.360 (0.345–0.373)
	MIMIC-CURB-65	0.660 (0.618–0.703)	0.965 (0.959–0.970)	1.000 (0.999–1.000)	0 (0–0.024)	0.965 (0.959–0.970)	-
	MIMIC-NEWS2	0.674 (0.630–0.718)	0.965 (0.959–0.970)	1.000 (0.999–1.000)	0 (0–0.024)	0.965 (0.959–0.970)	-
Invasive mechanical ventilation (IMV)	Mayo ^1^	0.736 (0.711–0.763)	0.870 (0.859–0.880)	0.932 (0.929–0.945)	0.321 (0.271–0.364)	0.924 (0.914–0.931)	0.389 (0.334–0.437)
	MIMIC	0.724 (0.711–0.735)	0.858 (0.853–0.862)	0.915 (0.907–0.923)	0.347 (0.333–0.360)	0.911 (0.905–0.917)	0.396 (0.382–0.407)
	MIMIC-CURB-65	0.649 (0.630–0.668)	0.814 (0.802–0.825)	1.000 (0.999–1.000)	0 (0, 0.005)	0.814 (0.802–0.825)	-
	MIMIC-NEWS2	0.700 (0.6810.720)	0.799 (0.787–0.811)	0.925 (0.916–0.933)	0.249 (0.220–0.280)	0.843 (0.832–0.855)	0.431 (0.386–0.477)
Non-invasive mechanical ventilation (NIMV)	Mayo ^1^	0.732 (0.718, 0.747)	0.663 (0.648–0.678)	0.673 (0.658, 0.687)	0.619 (0.602–0.634)	0.892 (0.880–0.901)	0.351 (0.336–0.365)
	MIMIC	0.708 (0.699–0.720)	0.645 (0.639–0.651)	0.651 (0.635–0.666)	0.636 (0.622–0.646)	0.880 (0.870–0.887)	0.378 (0.359–0.391)
	MIMIC-CURB-65	0.693 (0.677–0.708)	0.597 (0.583–0.612)	0.867 (0.847–0.886)	0.492 (0.474–0.509)	0.904 (0.889–0.918)	0.401 (0.382–0.420)
	MIMIC-NEWS2	0.724 (0.709–0.740)	0.658 (0.644–0.672)	0.727 (0.701–0.752)	0.631 (0.614–0.648)	0.855 (0.840–0.869)	0.436 (0.415–0.458)

Note: ^1^ Mayo Clinic internally validated prediction model. AUC = area under the receiver operating characteristic; Spec = Specificity; Sens = Sensitivity; NPV = negative predictive value; PPV = positive predictive value; CI = confidence interval; MIMIC-CURB-65 = MIMIC cohort using CURB-65 as prediction model; MIMIC-NEWS2 = MIMIC cohort using NEWS2 as prediction model.

## Data Availability

MIMIC-IV database, a publicly available critical care database, was used for this validation study. Access was granted after completion of the required training and data use agreement. We did not create any new data.

## Supplementary Materials

The following supporting information can be downloaded at: https://www.mdpi.com/article/10.3390/idr18040077/s1, Figure S1: Calibration plot by Age group; Figure S2: Calibration Plot for COPD; Figure S3: Calibration Plot for CHF; Figure S4: Variable importance.

## Abbreviations

CAP—community-acquired pneumonia; HFNC—high flow nasal cannula; NIMV—non-invasive mechanical ventilation; IMV—invasive mechanical ventilation; PSI—pneumonia severity index; CURB-65—confusion, urea, respiratory rate, blood pressure, and age 65 and older; ATS/IDSA-American thoracic society/Infectious disease society of America; NEWS, NEWS2—National Early Warning Score; MEWS—Modified Early Warning Score; SIRS—Systemic Inflammatory Response Syndrome; GBM–gradient boosting machine; MIMIC-IV—Medical Information Mart for Intensive Care IV; AUC—area under curve; NPV—negative predictive value; PPV—positive predictive value: CI- confidence interval: ICU- intensive care unit; SOFA- sequential organ failure assessment; ICD- international classification of diseases; IQR-interquartile range; CHF- congestive heart failure; COPD- chronic obstructive pulmonary disease.
