# Supplementary material for: Early Machine Learning-Based Identification of Hospitalized Patients at Low Risk of Respiratory Deterioration or Mortality in Community-Acquired Pneumonia: External Validation of a Multivariable Model
Source: Infect Dis Rep. 2026 Jul 25;18(4):77. doi: 10.3390/idr18040077 (PMC13512326; doi:10.3390/idr18040077)

Supplementary Figure S1- Calibration plot by Age group

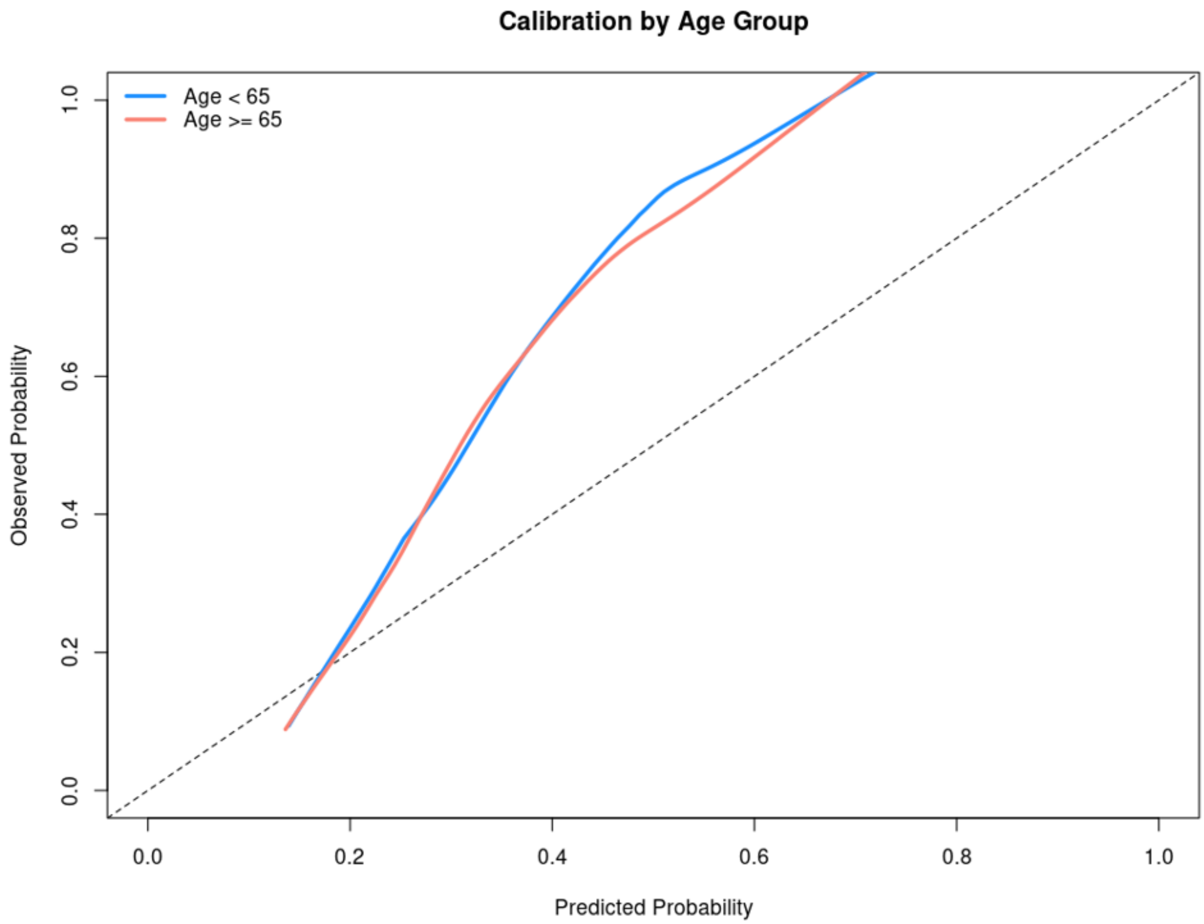

Supplementary Figure S2- Calibration Plot for COPD

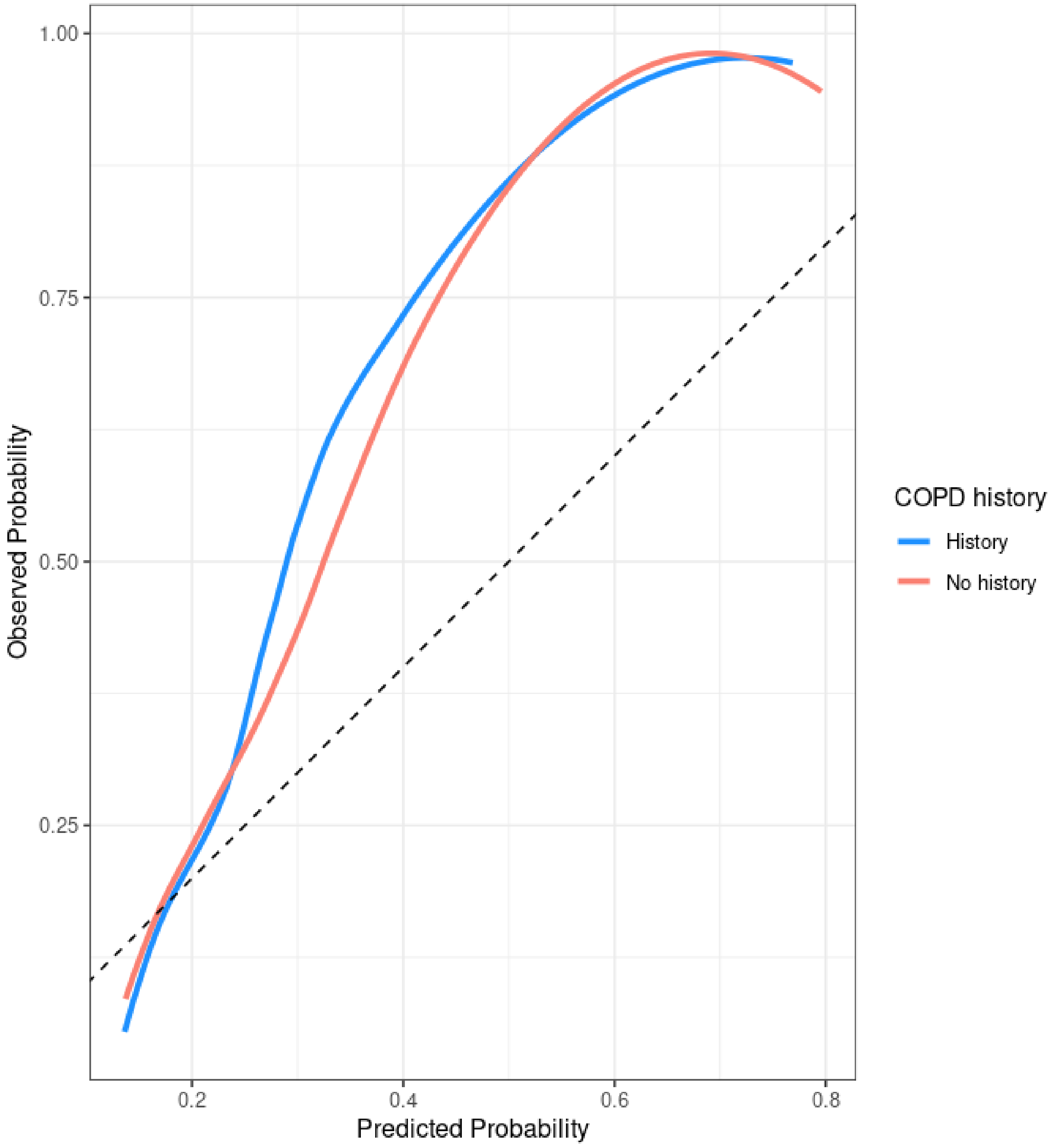

Supplementary Figure S3- Calibration Plot for CHF

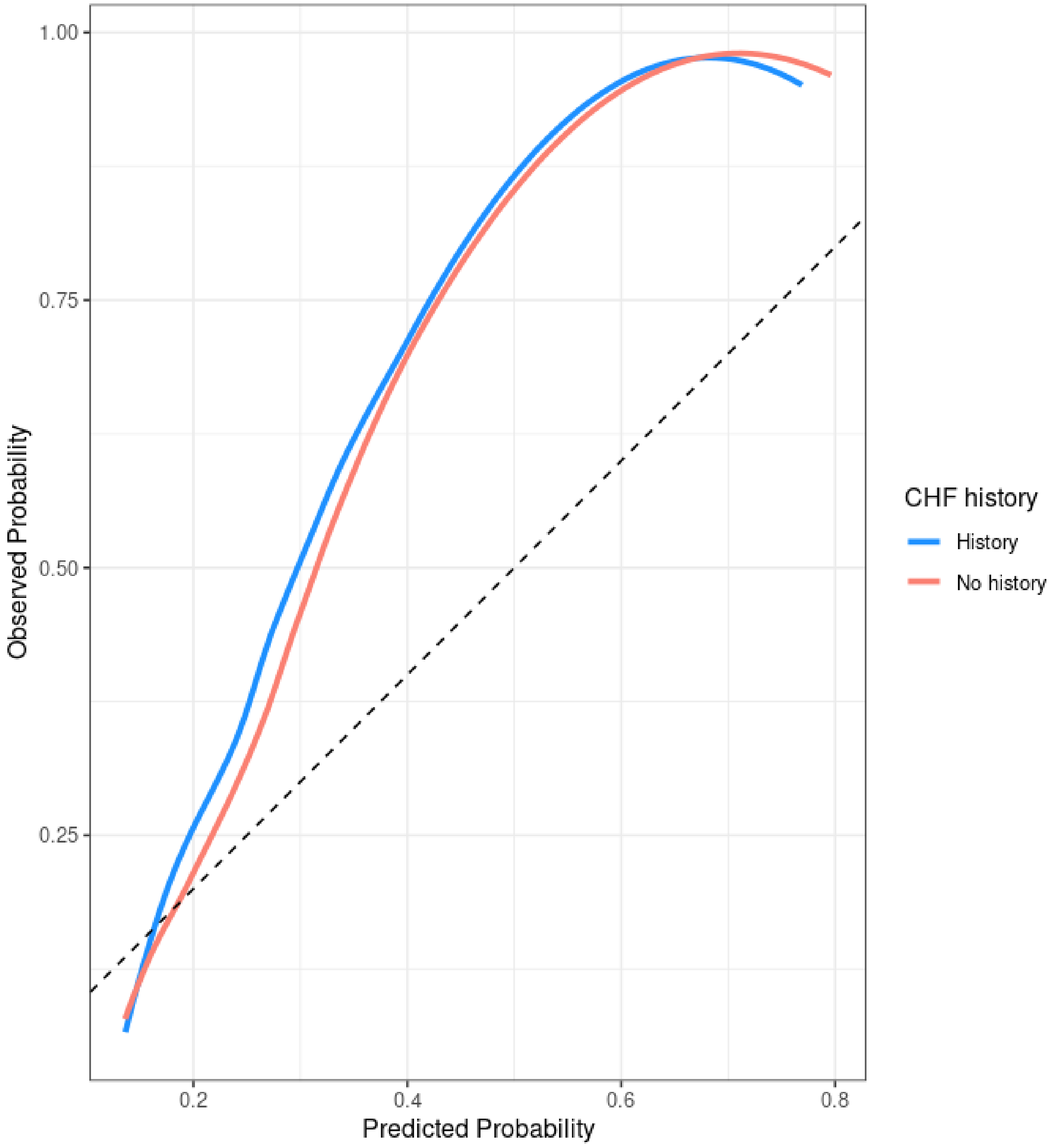

Supplementary Figure S4- Variable importance

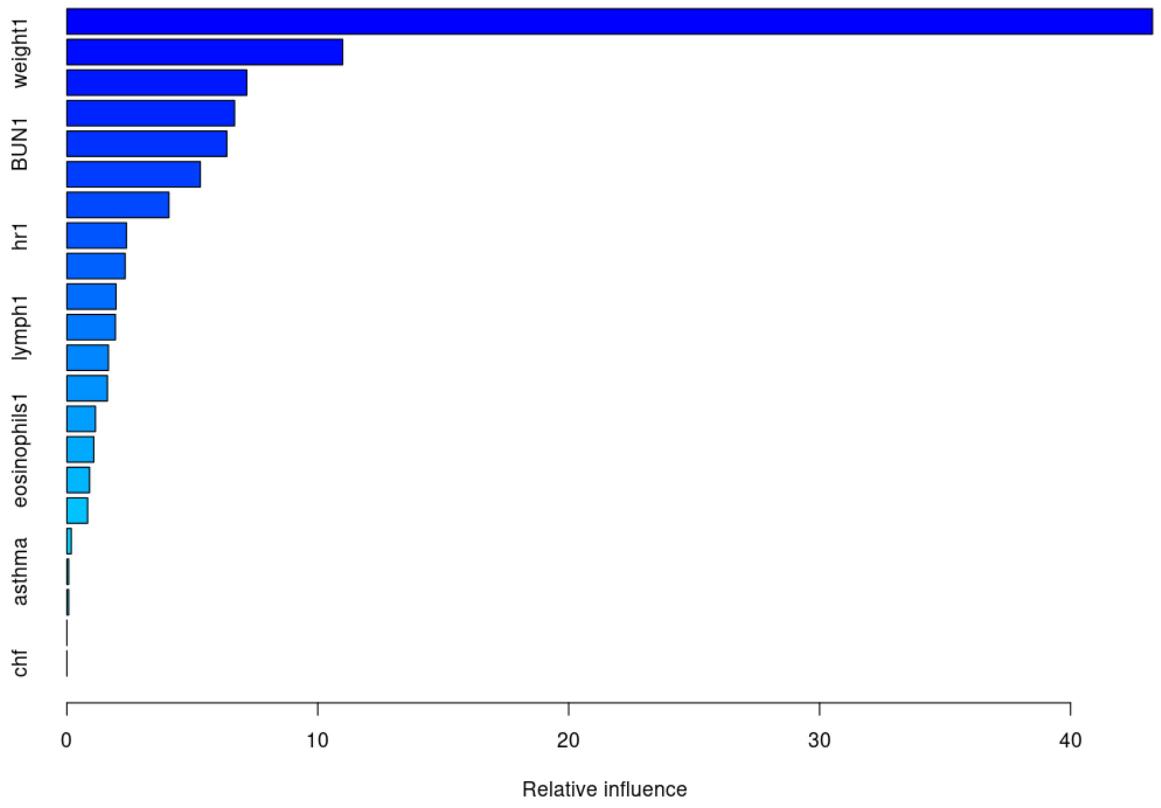

Supplement: Supplementary file 1 [file idr-18-00077-s001.zip › idr-4308691-supplementary.pdf]
